# Microfluidic-Based Droplet and Cell Manipulations Using Artificial Bacterial Flagella

**DOI:** 10.3390/mi7020025

**Published:** 2016-02-08

**Authors:** Yun Ding, Famin Qiu, Xavier Casadevall i Solvas, Flora Wing Yin Chiu, Bradley J. Nelson, Andrew deMello

**Affiliations:** 1 Institute for Chemical and Bioengineering, Department of Chemistry and Applied Biosciences, ETH Zürich, Vladimir Prelog Weg 1, 8093 Zürich, Switzerland; yun.ding@chem.ethz.ch (Y.D.); xavier.casadevall@chem.ethz.ch (X.C.S.); flora.chiu@chem.ethz.ch (F.W.Y.C.); 2 Institute for Robotics and Intelligent Systems, ETH Zürich, Tannenstrasse 3, 8092 Zürich, Switzerland; qiuf@ethz.ch

**Keywords:** microfluidic droplet, bio inspired microrobotics, helical microswimmer, artificial bacterial flagella, single droplet manipulating, motorized cell, artificial biological microorganism

## Abstract

Herein, we assess the functionality of magnetic helical microswimmers as basic tools for the manipulation of soft materials, including microdroplets and single cells. Their ability to perform a range of unit operations is evaluated and the operational challenges associated with their use are established. In addition, we also report on interactions observed between the head of such helical swimmers and the boundaries of droplets and cells and discuss the possibilities of assembling an artificial swimming microorganism or a motorized cell.

## 1. Introduction

**Figure 1:**
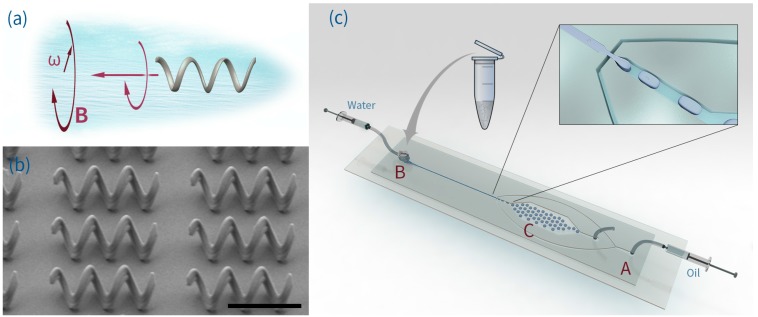
(**a**) Illustration of a helical ABF propelled by a rotating weak magnetic field B; (**b**) SEM image of a printed (and uncoated) MS array on a glass substrate (scale bar is 10 μm); (**c**) Schematic of the PDMS microdevice that contains a flow focusing structure for encapsulating ABFs in droplets. Port A serves as the oil inlet and port B is coupled to a chip-to-world connector, from which the aqueous suspension of ABFs is introduced.

Helical microswimmers represent a category of microrobotic tools recognized as highly promising solutions for biomedical applications including minimally invasive surgery and targeted drug delivery [1,2,3]. Indeed, their implementation and controlled actuation within *in vivo* systems has recently been reported for deep tissue analysis [4]. The basic structure of a helical microswimmer is inspired by flagellated bacteria, where thin, whip like appendages protruding from the cell body are used to move the bacteria towards nutrients and other chemo-attractants. Accordingly, such microswimmers are often termed artificial bacterial flagella (ABFs), and have been used to address the challenging issue of swimming within the low Reynolds number regimes typical on the microscale [5,6,7]. In contrast to chemically-propelled microswimmers [8,9] and artificial cilia constructed from electroactive polymers [10,11], magnetically actuated helical microswimmers rely on the application of magnetic fields for motion [12,13]. Put simply, ABFs move in fluid environments by translating rotational motion to translational motion under the application of low-strength rotating magnetic fields. Such an approach is especially advantageous since the swimmer can be powered remotely (without the need for an on-board fuel source) and manoeuvred in controllable and dynamic fashion (Figure 1a). Unsurprisingly, the development of control methods for ABF manipulations [7] and the characterization of their swimming properties [13,14,15] has been an active area of research, with recent efforts being focused on the addition of supplementary functional units (such as “micro-holders”, “micro-bars”, and “micro-rings”) for the manipulation and transport of microscale objects [16,17,18], developing magnetic composites exhibiting superior biocompatibility [19,20] and the functionalization of ABF surfaces to furnish them with novel properties [21,22,23]. Although, the fabrication of ABFs has been described in detail elsewhere [16,24], it should be noted that adoption of two-photon polymerization (2PP) techniques for micro-/nanostructure fabrication [25,26,27] has allowed the rapid realization of three-dimensional micro-/nanoscale structures in a variety of materials [28,29,30].

In principle, ABFs can be used for manipulating microscopic objects to realize specific chemical and biological operations in both *in vitro* and *in vivo* environments. Nevertheless, to date, ABFs (with dimensions below 100 µm) have only been used to manipulate “hard” objects under idealized experimental environments, such as deionized water [16,17]. Accordingly, their potential utility in many real biological systems remains an unresolved issue. Over the past two decades, microfluidic systems (commonly termed Lab-on-a-Chip devices) have become increasingly popular platforms in which to perform a wide range of chemical and biological assays and process a diversity “soft” biological entities (e.g., microdroplets, gel microparticles, and cells) [31,32,33,34]. The ability to produce, manipulate and process soft objects in a high-throughput manner is an important feature of microfluidic systems, and their utility in applications such as digital PCR [35,36] drug delivery systems [37], single cell analysis [38,39], nanomaterial synthesis [40,41], and the generation of artificial tissues [42,43] is well documented. To demonstrate the feasibility of using ABFs to manipulate soft materials in the context of wide range of chemical and biological applications, we herein explore the three highly relevant scenarios incorporating soft microdroplets and cells. Specifically, we assess the ability of ABFs to perform a range of unit operations on soft objects within microfluidic environments and evaluate the operational challenges associated with their use.

## 2. Experiment, Results and Discussion

### 2.1. Operation inside Microfluidic Droplet

To begin, we encapsulated ABFs within water-in-oil droplets generated using a microfluidic flow-focusing geometry and assayed the capacity for ABFs to swim within an isolated nL-volume compartment. Droplets (or segmented flows) produced within microfluidics offer significant advantages when performing high-throughput biological and chemical experimentation [44,45,46]. Briefly, fL-nL volume droplets can be generated at kilohertz frequencies, where each droplet acts as an isolated assay volume. Since droplet volumes are extremely small, a wide range of assays can be performed using reagent volumes 6–9 orders of magnitude less than typically used in macroscale platforms. Additionally, the use of an immiscible continuous phase to encapsulate each droplet ensures minimal reagent interaction with channel walls and the removal of residence time distributions.

Various types of small samples (down to the single molecule or cell level) can be encapsulated or dosed into microdroplets for a range of applications [47,48]. Interestingly, Brouzes *et al.* recently encapsulated micron-sized super-paramagnetic beads to extract desired molecules from droplets in a segmented flow [49]. Although the motion of such magnetic beads can somewhat be controlled, locomotion at small scales (*cf.* the scallop theorem) cannot easily be achieved with these systems [50]. This shortcoming can however be readily overcome through the use of robotic ABFs.

For the current experiments, ABFs (16 μm in length and 5 μm in diameter) were fabricated by 2PP-based 3D laser lithography. The surface of the formed ABFs was then coated with a 50 nm Ni film and a 5 nm Ti film. The Ni layer provides the ABF with its magnetic properties and the Ti layer (autoxidized to titanium dioxide) increases biocompatibility [51]. Figure 1b presents an SEM image of a printed ABF array on a glass substrate. ABFs were harvested by ultrasonic detachment from the substrate and prepared as a suspension for subsequent use. A polydimethylsiloxane (PDMS) chip incorporating a flow focusing droplet generator was used to encapsulate single ABFs inside aqueous droplets (single-digit nL). Figure 1c illustrates the fluid connections and generic chip structure used to form aqueous droplets and encapsulate ABFs. Specifically, regions A and B define the oil (FC-40) and water inlets, respectively, and region C contains a storage chamber for droplet incubation and observation. To ensure that ABF losses within the connecting tubing (via adhesion of ABFs onto the plastic surfaces) are minimized, a chip-to-world connector [52] is used in region B allow direct injection of a small but defined volume of the ABF suspension. After encapsulation, ABFs were controlled using an in-house electromagnetic control system that generates a rotational magnetic field, which can be monitored and varied in real-time. Detailed information regarding the fabrication, materials, sample preparation and operational protocols can be found in Appendix 1, Appendix 2 and Appendix 3.

**Figure 2:**
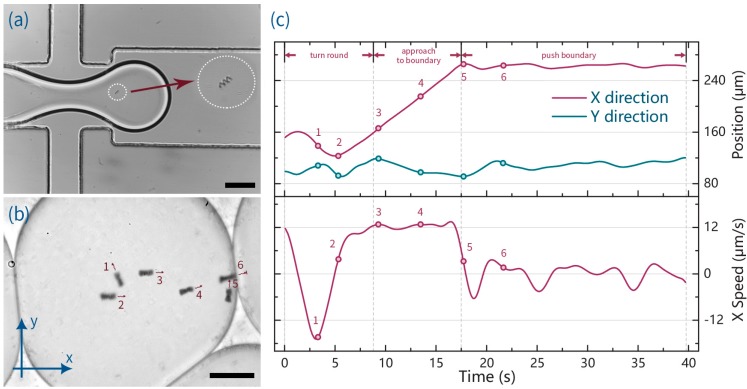
(**a**) Image showing the formation of an ABF-containing droplet. The ABF is shown within the white dotted circle; (**b**) A time-lapse image showing controlled locomotion of an ABF within a droplet. The magnetic field is 90 Hz and 9 mT; (**c**) Variation of ABF position and speed for experiment shown in (b). The upper plot illustrates the *x*, *y* position as a function of time and the lower plot shows the variation of velocity in the *x* direction. It is noted that when the ABF reaches the droplet boundary (at 17.5 s), the net displacement and average speed in *x* are both zero. Scale bars correspond to 100 μm.

Figure 2a illustrates the encapsulation of an ABF inside an aqueous droplet at a microfluidic flow focusing geometry. Without additional modification of the droplet generation process, ABFs could be loaded into droplets in a high-speed and in a direct manner [53,54], with droplet occupancies following a Poisson distribution [36,55]. After formation, droplets were motivated to the storage chamber, where ABF swimming performance could be evaluated. Figure 2b and Video S1 show a pair of ABFs (in a “chain configuration” [56]) swimming within the confines of a 5 nL droplet in response to a dynamically varying electromagnetic field. An analysis of both the position and speed of the swimmer is presented in Figure 2c. It is observed that the swimmer can be steered in any desired direction through variation of the applied electromagnetic field, and moreover remains within the confines of the droplet, even when forced to swim against the droplet boundary (for example at 17.5 s). In this regard, the interfacial tension associated with the curved droplet boundary generates a pressure difference between the inside and the outside of the droplet (the so-called *Laplace* pressure) [57,58], which is given by:

(1) $$\Delta P=\gamma \left(\frac{1}{R_{1}}+\frac{1}{R_{2}}\right)$$

where, γ is the surface tension and *R*_1_ and *R*_2_ are two principal radii of curvature. Since droplets are squeezed between bottom and top chip layers, *R*_1_ and *R*_2_ can be considered to be half the channel height and the radius of a droplet can be extracted from the brightfield image. Using the surface tension values for FC-40 with 3% (*w*/*w*) PFPE-PEG surfactant and a typical pushing pressure of 200 pN [17,59,60], we can estimate the equivalent radius for the push effect by an ABF to be larger than 1000 μm. Accordingly, for the current system (where the average droplet radius is 100 μm) the increased proportion of localized curvature will be less than 1/10, and, thus, droplet deformation by an ABF can be considered negligible. Indeed, as long as an ABF has no wetting affinity for the droplet/continuous phase boundary, it will remain inside the droplet under all conditions. Accordingly, we expect that the integration of ABFs with such droplets offers new opportunities for the direct manipulation of droplet contents, such as cells, nanomaterials, bacteria and other microorganisms [61].

### 2.2. Manipulation of Microdroplets

The ability to controllably propel and manipulate individual droplets is an additional and potentially important unit operation [62,63,64,65]. Indeed, such operations have been used to fabricate artificial tissues [43] and are indispensable in digital microfluidic applications [66]. However, it is noted that, to date, digital microfluidic platforms have been unsuccessful in robustly manipulating sub-nL droplets [67]. Accordingly, we assessed the ability of ABFs to individually transport picoliter-sized droplets. Water droplets of a size comparable to our ABFs were generated using a 5 μm wide flow-focusing geometry and transferred to a reservoir containing ABFs.

**Figure 3:**
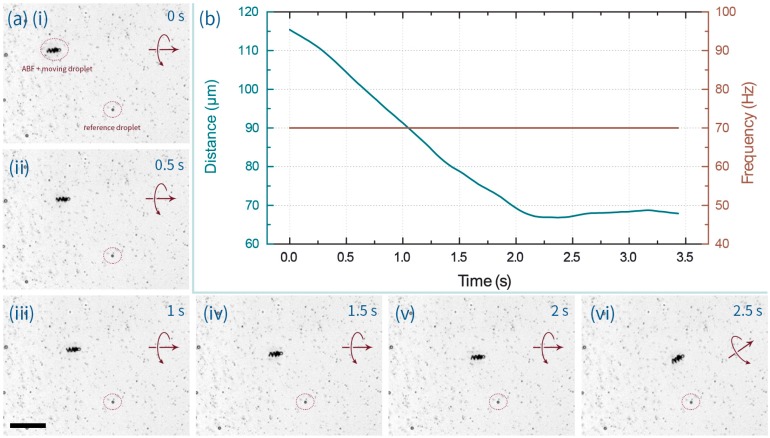
(**a**) Time lapse images of an ABF transporting a microdroplet within a 25 μm deep PDMS chamber, under the application of a 70 Hz, 7 mT magnetic field. The scale bar corresponds to 50 μm. (**i**–**vi**) Time lapse images at 0.5 s intervals. (**b**) Assessment of the relative distance between the transported droplet and a reference droplet as a function of time.

**Figure 4:**
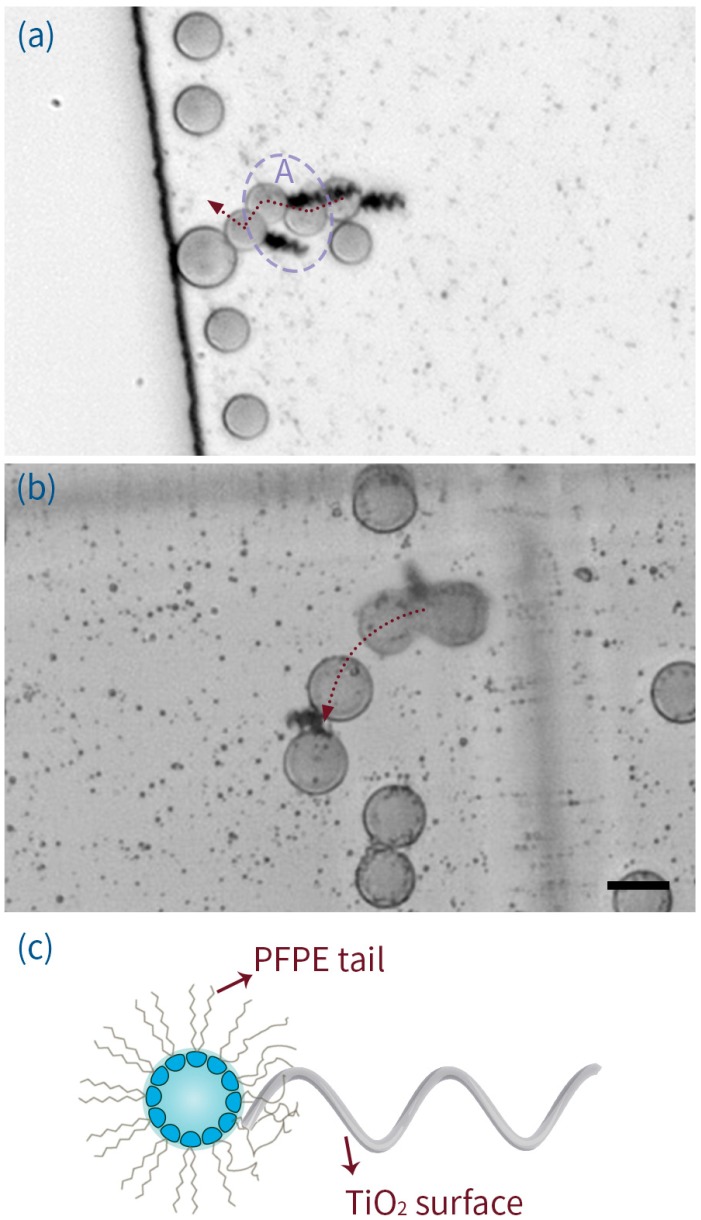
(**a**) A time-lapse image showing the transportation of a large droplet by an ABF across a 25 μm deep PDMS chamber, under the application of a 70 Hz, 7 mT magnetic field. Note the “swinging” behavior observed in region A. (**b**) A time-lapse image showing two large droplets being simultaneously transported along a 100 μm deep PDMS chamber. (**c**) Schematic explanation of the “soft” adhesion observed between surfactant coated droplets and the tip of an ABF. The scale bar corresponds to 20 μm and the straight black line defines the chamber wall.

**Figure 5:**
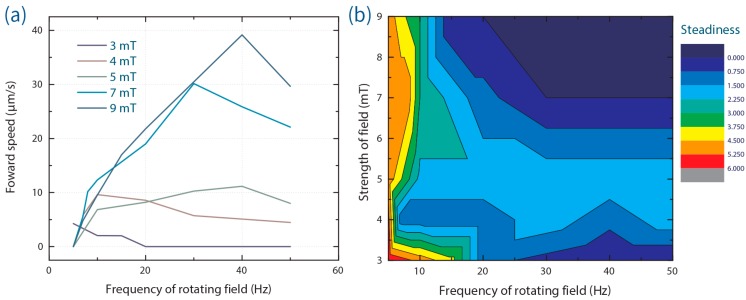
Swimming characterization in FC-40 oil (containing 3% *w*/*w* PFPE-PEG-PFPE tri-block surfactant) along a 100 μm deep PDMS chamber. (**a**) Forward swimming speed of ABFs as a function of magnetic field strength and rotation frequency. (**b**) Evaluation of the steadiness of swimming. Here, higher values represent reduced “steadiness”. “Wobbling” and “shifting” are two deleterious factors used to assay the swimming performance of ABFs (the assay methodology is provided in Appendix 4). The combination of these data provides the range of operational parameters that deliver stable swimming performance.

Data presented in Figure 3 demonstrate that ABFs are competent in transporting aqueous microdroplets. In addition to factors such as rotation frequency of the magnetic field, fluid viscosity [13] and payload size, propelling performance (*i.e.*, speed) is largely controlled by the friction between the droplet and the reservoir walls. Indeed, three main situations could be identified. First, when operating within “open” reservoirs (e.g., a plastic tank, 2 cm long, 1 cm wide and 0.5 cm deep), both droplets and ABFs could freely float on the fluorinated continuous phase (of higher density) and droplet transport was facile. For example, Video S2 shows a droplet being propelled by an ABF less than half its size, even against the direction of a residual convective flow. Second, in a fully enclosed (PDMS or PMMA (polymethyl methacrylate)) chambers, droplets that were large enough to be squeezed between the top and bottom walls, or droplets that displayed some degree of affinity for the chamber walls, could not be maneuvered (see Video S3). Finally, droplets with diameters less than the chamber height and no apparent affinity for the channel walls could also be smoothly transported within enclosed chambers. In this regard, Figure 3a shows a sequence of images of a small droplet being transported along a PDMS chamber 25-μm deep (also in Video S4). In addition, Figure 3b describes the relative position of a reference droplet over time. Droplet size (a proxy of payload) was also observed to affect the delivery rate. Figure 4a illustrates the propulsion of a larger droplet within an identical PDMS chamber and using the same operational field parameters (70 Hz and 7 mT). It was observed that the larger droplet could be maneuvered at a speed of 7.5 μm/s, which is approximately 4 times slower than the achievable velocity of the droplet described in Figure 3 (30.3 μm/s). This indicates that the payload (in this case volume) of the sample to be transported is an important factor in defining ABF-mediated delivery. It should also be noted that ABF swimming performance within the FC-40 fluorinated oil (dynamic viscosity = 4.1 cP) closely mirrored that within aqueous environments (e.g., deionized water; dynamic viscosity, 0.89 cP), although with reduced swimmer velocities [16,21]. The characterization of ABF swimming capabilities in FC-40 is shown in Figure 5a,b.

An interesting observation was the presence of “soft” adhesion forces between the TiO_2_-ABFs and the surface of both, droplets and cells. This unexpected interaction is instrumental in aiding reliable manipulation of soft particles. Figure 4a (area A) and Video S5 clearly illustrate the effect, where a droplet is successfully delivered to a larger one despite the ABF undergoing a brief period of destabilization. Adhesion also makes it possible for an ABF to transport two droplets simultaneously (Figure 4c and Video S6). We suspect that this phenomenon is caused by the adsorption and entanglement of the perfluoropolyether surfactant tails on the TiO_2_ surface (Figure 4c), a mechanism similar to that reported and characterized by Walczak *et al.* [68]. In principle, such adhesion effects could be used to more closely associate ABFs with droplets (or cells), to create biosynthetic, propulsion assemblies, mimicking naturally swimming microorganisms, such as *E.coli* and spermatozoa.

### 2.3. Motorized Cell

Single cell manipulation via magnetic microrobotics has previously been realized through the use of a U-shaped microtransporter [69]. It is noted however, that this tool is limited to transport on surfaces since it lacks the 3D locomotion capabilities of ABFs. To this end, we assessed the feasibility of assembling ABFs on individual cells for propulsion purposes. Human B lymphocytes (that identify pathogens when surface antibodies bind to a foreign antigen) are key components within the human immune system, and thus interesting candidates for “motorization”.

**Figure 6:**
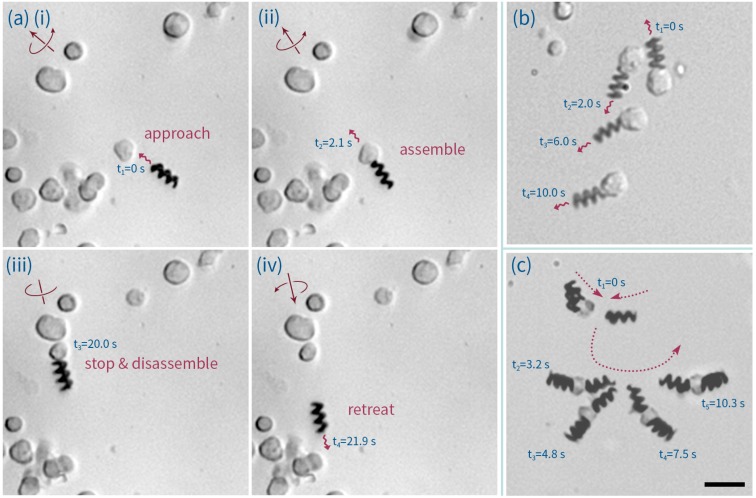
(**a**) The process by which a motorized cell is assembled and disassembled. In (i) and (ii) an ABF approaches the desired cell, contacts it and assembles. The B-cell-ABF assembly then moves forward following the direction of the applied rotating magnetic field (iii and iv). To disassemble, the rotating direction of magnetic field is simply reversed, allowing the ABF to move back and detach from the cell. (**b**) A time-lapse image of a cell being dragged. (**c**) A time-lapse image of the process of forming an ABF-cell-ABF configuration. The scale bar corresponds to 20 μm.

We successfully assembled and disassembled B cells onto ABFs (a description of the treatment of B cells in these experiments can be found in the Appendix 5). Figure 6a and Video S7 show such a process. Here, it can be seen that an ABF is steered toward a Human B lymphocyte cell at a velocity of 8.7 μm/s (accelerated from standing). On contact, the cell/ABF assembly continues towards the target cell at a velocity of 2.9 μm/s. After the ABF has delivered the cell to the desired location, it then disassembles and retreats at a velocity of 18.1 μm/s. Compared to data associated with droplet transport, assembly and motivation of Human B lymphocyte cells is associated with a stronger entanglement effect with the ABFs, which is likely to be due to the richness and complexity of the biological cell membrane (which will contain a wide variety of cell receptors and adhesion proteins). In some cases, this interaction was so strong that cells could be dragged by the ABF (Figure 6b and Video S8) to form tandem cluster configurations, such as those shown in Figure 6c (and Video S9). Observation of such motorized cells over a period of 1 h of operation revealed no observable changes in morphology. Moreover, viability assays of cells actuated by ABFs indicated no change in cell mortality when compared to control samples (details are provided in the Appendix 6).

## 3. Conclusions

In conclusion, we have explored and validated for the first time the manipulation of soft materials by magnetically actuated, helical microswimmers. Such swimmers were shown to exhibit uniform swimming performance in a variety of fluid environments (including deionized water, cell growth media and fluorinated oils). Significantly, precise and controlled ABF locomotion was demonstrated for the first time within confined fluid volumes (such as water-in-oil droplets). Furthermore, the intimate interaction between ABFs and both microdroplets and Human B lymphocyte cells was leveraged to allow controlled assembly and disassembly of objects with complex configurations. We recognize that such droplet/cell-ABF assemblies could be used to mimic biological microorganisms, while “motorized” B cells have the potential to enable targeted immunotherapies *in vivo*. Accordingly, we believe that ABFs represent a promising engineering tool for microfluidic droplet and cell-based experimentation at the single unit level.

## Figures and Tables

**Figure A1:**
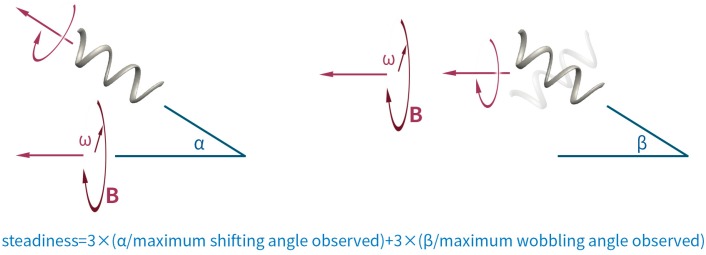
Calculation of the steadiness variable. The left image describes the shifting phenomenon and the right image the wobbling phenomenon.

**Figure A2:**
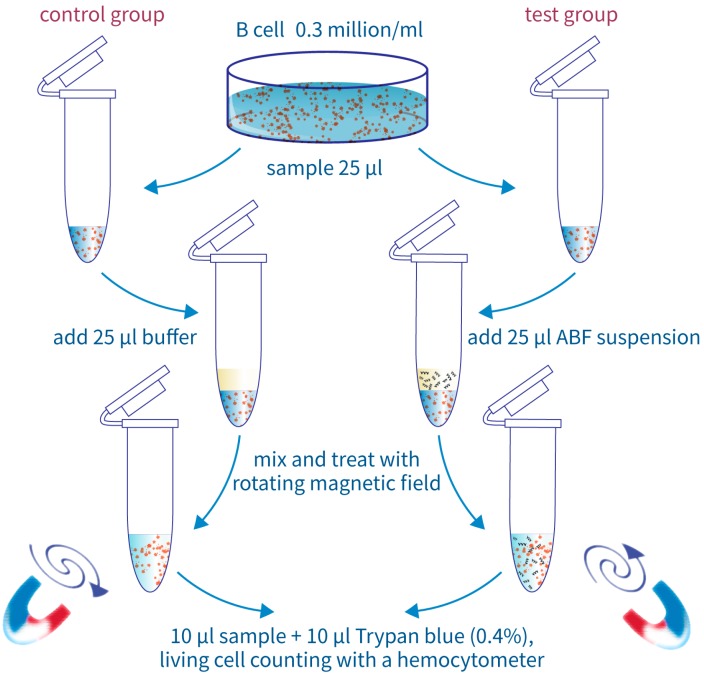
Protocol for testing cell viability.

## Supplementary Materials

The following are available online at http://www.mdpi.com/2072-666X/7/2/25/s1. Nine video clips are combined into a single one with a title for each section. Video S1: manipulation of microswimmers within a droplet, Video S2: targeted delivery of a droplet in an uncapped tank, Video S3: a droplet that wets the surface and cannot be moved, Video S4: transportation of a droplet within a PDMS chamber, Video S5: adhesion between a droplet and a microswimmer, Video S6: simultaneous movement of two droplets, Video S7: assembling and disassembling a motorized cell, Video S8: a microswimmer dragging a cell, Video S9: formation of a microswimmer-cell-microswimmer assembly.

## Appendix

### 1. ABF Fabrication and Suspension Preparation

A 3D laser lithography system (Photonic Professional, Nanoscribe GmbH, Eggenstein-Leopoldshafen, Germany) was used to print all artificial bacterial flagella used in the current work. A negative-tone photoresist (IP-L 780, Nanoscribe GmbH, Germany) optimized for two-photon polymerization was used to achieve high resolution combined with simplicity of use. Briefly, processing consisted of writing, developing and drying the photoresist, followed by deposition of two metal layers (50 nm Ni and 5 nm Ti) by physical vapour deposition (Evaporation Plassys II, PLASSYS, Marolles-en-Hurepoix, France). A detailed description of the complete process can be found elsewhere [16]. To form a fluid suspension of the microswimmers, the formed arrays were detached from a glass substrate via sonication. This involved removal of excess regions of glass substrate and insertion of the remaining substrate into a 1.5 mL centrifuge tube (Eppendorf^®^ LoBind, Sigma-Aldrich Chemie GmbH, Buchs, Switzerland). This was followed by addition of 0.2 mL of deionized water. Sonication at 40 kHz for 15 min resulted in the detachment of more than 95% of the microswimmers. Microwimmers intended for use in fluorinated oil (Fluorinert^®^ FC-40, Sigma Aldrich, Switzerland) were dehydrated under vacuum overnight before being suspended in FC-40 (containing surfactant).

### 2. PDMS Chip Fabrication and ABF Encapsulation

PDMS (Sylgard^®^ 184, Dow Corning, Biesterfeld Spezialchemie GmbH, Hamburg, Germany) microdevices were fabricated using standard soft lithographic methods. Details of the entire procedure are provided elsewhere [70]. The top structured PDMS layer was subsequently bonded to a flat bottom PDMS layer by treatment in an oxygen plasma. Finally, the whole assembly was contacted with a glass microscope slide to provide mechanical strength.

To create water-in-oil droplets, an oil phase FC-40 containing 3% (*w*/*w*) of a PFPE-PEG surfactant (EA-Surfactant, RainDance Technologies) was combined with a deionized water stream at a standard flow focussing geometry, with hydrodynamic motivation being provided by syringe pumps (neMESYS Low Pressure Dosing Module, Cetoni, Korbußen, Germany). To encapsulate single microswimmers within such droplets, microchannels were initially filled with the oil phase to expel gas bubbles and prevent wetting of the discrete phase. 20 μL of the ABF suspension was then added through the chip-to-world connector. After delivering oil (20 μL/min) and water (10 μL/min) for a period of 90 s, a stable segmented flow of droplets (which were either empty or containing a single microswimmer) was achieved.

### 3. Magnetic Control System

The primary part of the magnetic control system consists of three pairs of electromagnetic coils oriented along the Cartesian coordinate systems of three-dimensional space. Operational variables include magnetic intensity (up to 10 mT), rotating frequency (up to 200 Hz), pitch angle and yaw angle. A detailed description of the complete system can be found elsewhere [7,16].

### 4. Method to Assess Swimming Steadiness

To quantitatively evaluate steadiness we assign relative values to two undesirable phenomena that arise during ABF operation; *shifting* (when the ABF trajectory deviates from the direction induced by the magnetic field) and *wobbling* (when the ABF wobbles but preserves its axial direction). If neither shifting nor wobbling are present, a score 0 is given for each respective parameter; conversely, a score of 3 is given for the maximum shifting or wobbling angles observed. A final score quantifying steadiness for any ABF is calculated using the relationship shown in Figure A1.

### 5. Treatment of Biological Cells

Human B lymphocyte cells were handled and processed according to the following protocol. Prior to experimentation, cells were cultured in RPMI 1640 growth medium (Life Technologies Europe B.V., Zug, Switzerland) supplemented with 10% (*v*/*v*) heat-inactivated fetal bovine serum (Invitrogen, UK), 2 mM L-glutamine (Invitrogen, UK), 50 U/mL penicillin and 50 µg/mL streptomycin (Invitrogen, UK). For experiments, cells were washed once in calcium- and magnesium-free phosphate-buffered saline (Invitrogen, UK) and counted with a haemocytometer. Cells were then re-suspended in calcium- and magnesium-free phosphate-buffered saline with 2% (*v*/*v*) heat-inactivated fetal bovine serum in the desired concentration and volume.

To ensure that microswimmers did not stick to exposed microchannel surfaces, a thin layer of PDMS (100 μm in height) was deposited on a microscope glass slide (76 × 26 × 1 mm; Menzel-Glaser, Braunschweig, Germany) via spin-coating and followed by the curing at 70 °C in oven for 1 h. Subsequently a sheet of SA8S-0.5-SecureSeal (Grace Bio-Labs, Bend, OR, USA) was placed over the PDMS layer to form 8 chambers (7 mm wide, 7 mm long and 0.5 mm deep).

### 6. Cell Viability Assessment

The cell viability assay follows the protocol described in Figure A2, with only dead cells being stained. Results shown in Table A1 indicate minimal variation in viability over a 2 h timescale.

**Table A1:** Cell viability assay results.

Time	Living Cell Proportion (Control)	Living Cell Proportion (Test)
15 min	97.3%	98.6%
30 min	96.2%	97.6%
60 min	96.1%	96.3%
120 min	94.0%	94.5%
